# Challenges in the Diagnosis and Management of Triosephosphate Isomerase Deficiency: A Case Report

**DOI:** 10.3390/reports8030162

**Published:** 2025-09-01

**Authors:** Carolina Ramos, Inês Pereira, Joana Coelho, Patrícia Dias, Patrícia Lipari Pinto, Anabela Ferrão, Rosário Ferreira

**Affiliations:** 1 Pediatric Department, Hospital de Santa Maria, Unidade Local de Saúde de Santa Maria, 1649-035 Lisbon, Portugal; ines.a.pereira@ulssm.min-saude.pt; 2 Neuropediatric Unit, Pediatric Department, Hospital de Santa Maria, Unidade Local de Saúde de Santa Maria, 1649-035 Lisbon, Portugal; joana.coelho@ulssm.min-saude.pt; 3 Medical Genetics Service, Pediatric Department, Hospital de Santa Maria, Unidade Local de Saúde de Santa Maria, 1649-035 Lisbon, Portugal; patriciaalmeidadias@gmail.com; 4 Metabolic Unit, Hereditary Metabolic Disease Reference Center, Pediatric Department, Hospital de Santa Maria, Unidade Local de Saúde de Santa Maria, 1649-038 Lisbon, Portugal; ppinto3@edu.ulisboa.pt; 5 Haematology Unit, Pediatric Department, Hospital de Santa Maria, Unidade Local de Saúde de Santa Maria, 1649-035 Lisbon, Portugal; anabela.ferrao@ulssm.min-saude.pt; 6 Pediatric Respiratory Unit, Pediatric Department, Hospital de Santa Maria, Unidade Local de Saúde de Santa Maria, 1600-028 Lisbon, Portugal; rosario.ferreira@ulssm.min-saude.pt

**Keywords:** case report, haemolytic anaemia, neuromuscular dysfunction, rare disease, respiratory failure, triosephosphate isomerase deficiency

## Abstract

**Background and Clinical Significance**: Triosephosphate isomerase (TPI) deficiency is a rare autosomal recessive metabolic disorder caused by a pathogenic variant in the *TPI1* gene. It is characterised by chronic haemolytic anaemia, progressive neuromuscular dysfunction, and reduced life expectancy. Patients typically present with symptoms in the first few months of life, including muscle weakness, ataxia, and recurrent respiratory infections. Diagnosis is confirmed by genetic testing, and management is generally symptomatic as no treatment is available. **Case Presentation**: We describe the case of an infant diagnosed with TPI deficiency in the context of haemolytic anaemia with progressive neurological deterioration and respiratory failure. **Conclusions**: This case illustrates the complexity of the disease and highlights the importance of early diagnosis and contributes to the limited literature by providing a detailed clinical description and highlighting the diagnostic challenges associated with this condition. Beyond its clinical relevance, this report emphasises the potential role of personalised medicine in the management of TPI deficiency. Early identification of specific genotypes may inform prognosis and guide individualised supportive strategies. As knowledge of the molecular underpinnings of TPI deficiency expands, opportunities may emerge for targeted therapeutic approaches tailored to patient-specific characteristics.

## 1. Introduction and Clinical Significance

Triosephosphate isomerase (TPI) deficiency is a rare autosomal recessive disorder associated with progressive neurological impairment [1]. This condition was first described in the 1960s and is caused by pathogenic variants in the *TPI1* gene (NM_000365.6) [2]. The deficiency of this enzyme results in the disruption of the glycolytic pathway, leading to the accumulation of toxic intermediates and dysfunction of cellular energy [3]. The disorder primarily presents as early-onset chronic haemolytic anaemia, which is often accompanied by progressive neurodegenerative symptoms, including muscle weakness, ataxia, developmental delays and recurrent bacterial infections [4]. Furthermore, patients may develop diaphragmatic paralysis necessitating non-invasive ventilation (NIV), and cardiomyopathy.

Despite the severity of the disease, fewer than 50 cases have been documented worldwide, likely due to underdiagnosis and the fatal nature of the condition [5]. In Portugal, no registry data or published cases are available. The literature is scarce and primarily limited to individual case reports or small series [5,6,7,8], which hinders a broader understanding of the condition. This context underscores the need for increased awareness and further research into TPI deficiency, particularly in improving diagnostic strategies and therapeutic options.

Given the heterogeneous clinical presentation and complex diagnostic journey, TPI deficiency exemplifies the need for personalised medicine approaches in rare metabolic disorders. As the molecular basis of the disease becomes better understood, opportunities arise for genotype-specific interventions and novel treatment approaches adapted to individual clinical profiles.

## 2. Case Presentation

A 4-month-old girl presented to the emergency department with a three-day history of increased work of breathing.

On evaluation, significant tachypnea (90 breaths/min), with adequate O_2_-saturation (>96%); on auscultation, lung sounds were present with no abnormal sounds. The liver and spleen were not palpable below the costal margin. Neurological findings were significant for axial and peripheral hypotonia, with symmetrical distal brachial predominance paresis. The infant showed no tongue fasciculations, maintained excellent eye contact and smiling, and had normal and symmetrical deep tendon reflexes (DTR). There was no regression of developmental milestones. Low-flow oxygen therapy and nasogastric tube feeding were initiated for respiratory comfort.

Her medical history is significant for neonatal jaundice, needing phototherapy and a prior hospital admission at 2 months of age due to Respiratory Syncytial Virus (RSV) bronchiolitis and anaemia (haemoglobin 6.5 g/dL), requiring blood transfusion support. Her growth and development were normal. The patient was born to non-consanguineous parents, with no family history of neuromuscular disorders, anaemia, or metabolic conditions.

### 2.1. Investigations

This case report was conducted through the retrospective analysis of clinical data obtained during routine patient care, following parental informed consent. Diagnostic investigations were performed as per standard clinical guidelines.

Upon admission, a chest X-ray was performed, showing no significant abnormalities, along with blood tests showing anaemia (haemoglobin 9.6 g/dL, mean corpuscular volume 92 fL, reticulocytes 10.4%), without substantial increase in inflammatory markers (leukocytes 15.600 × 10^9^/L, C-reactive protein < 0.6 mg/L). Both the electrocardiogram and echocardiogram showed no anomalies. Due to the child’s distal motor weakness and hypotonia, an electromyography (EMG) was performed, which showed no anomalies. In this context, brain and cervical Magnetic Resonance Imaging (MRI) was conducted, documenting ectopic neurohypophysis, without other alterations. Endocrinological evaluation, specifically regarding the hypothalamic-pituitary axis, was normal. Creatine kinase levels were within the normal range. An abdominal ultrasound showed bilateral diaphragmatic hypomotility.

An extensive metabolic panel, which included vitamin B12, folate, homocysteine, amino acid chromatography, organic acids, and redox potential, was performed, all of which were normal. Acylcarnitine profile was markedly unusual, revealing increased levels of several short-chain species: C2 (acetylcarnitine) at 57.12 µM, C3 (propionylcarnitine) at 8.21 µM, and C5 (isovalerylcarnitine) at 0.38 µM, all above age-specific reference ranges. Notably, the elevation of C3 has been described in TPI deficiency and may reflect secondary metabolic perturbations due to impaired glycolysis.

The direct antiglobulin test (DAT) was negative. The enzyme assays for glucose-6-phosphate dehydrogenase and pyruvate kinase deficiency, membrane studies, and haemoglobin electrophoresis showed no abnormalities.

Genetic testing using a neuromuscular panel returned inconclusive, and a dysmorphology examination was negative for minor dysmorphisms or anomalies.

Therefore, trio whole-exome sequencing (WES) was performed, including analysis of the mitochondrial genome, revealing two heterozygous missense variants in the *TPI1* gene, in *trans*, both classified as likely pathogenic: c.130 C > T, p. (Pro44Ser) and c.556 C > T, p. (His186Tyr). This result establishes the diagnosis of triosephosphate isomerase deficiency. WES including mitochondrial genome analysis was conducted at Synlab using Twist Bioscience capture and library preparation, followed by sequencing on an Illumina platform with >98% of bases covered at 20x. Bioinformatic analysis aligned reads to the GRCh37/hg19 reference genome, and variant classification followed American College of Medical Genetics and Genomics (ACMG) guidelines. All pathogenic or likely pathogenic variants were confirmed by Sanger sequencing.

### 2.2. Treatment

Throughout the hospital stay, she experienced significant respiratory distress, needing nasal NIV, almost continuous, requiring supplemental oxygen therapy (O_2_ 0.5−2 L/min) during NIV pauses. She was fed by nasogastric tube with good tolerance and started oral feeding with positive results. During her stay, she also started a physical rehabilitation programme. She was discharged in a clinically stable condition with NIV support and supplemental oxygen therapy for the periods without NIV.

### 2.3. Outcome and Follow-Up

Currently, at the age of 13 months, the patient maintains extensive NIV dependence for more than 16 h/day. She has acquired some motor skills, such as sitting independently, gross grasp, object transfer, bringing hands to mouth, and partial rolling. Neurological examination reveals distal paresis in the upper limbs, difficulty with hand dorsiflexion, and mild atrophy of the interossei muscles. Under close monitoring, her haemoglobin levels stabilised (haemoglobin 9 g/dL) without further transfusion. She has maintained physical medicine and rehabilitation therapy (3 times per week), which has been integral in addressing her motor and developmental needs. She also has Speech Therapy once weekly and Occupational Therapy once weekly.

## 3. Discussion

We reported a patient with haemolytic anaemia and neuromuscular dysfunction, with a genetic diagnosis compatible with triosephosphate isomerase deficiency.

Triosephosphate isomerase is a crucial glycolytic enzyme responsible for the interconversion of the 3-carbon sugars dihydroxyacetone phosphate (DHAP) and glyceraldehyde−3-phosphate (G3P). This enzyme is essential for the breakdown of DHAP and the net production of adenosine triphosphate (ATP) through anaerobic glucose metabolism. TPI deficiency is a rare autosomal recessive disorder of infancy and childhood, resulting from several variants. The most common genotype is the E105D homozygous variant in *TPI1* gene [9]. This disease is classified as a glycolytic enzymopathy, with a limited understanding of its underlying pathogenesis. It has a broad spectrum of clinical presentations, which may overlap with many diseases**.** Clinical features usually include haemolytic anaemia, progressive neuromuscular dysfunction, and increased susceptibility to infection with specific pathogenic variants. Less common symptoms of TPI deficiency include cardiomyopathy and seizures, which add to the complexity of the disease presentation. Studies using TPI-deficient mouse models have provided insight into the underlying mechanisms contributing to the neuromuscular symptoms. Key factors include neurodegeneration in the brain, alterations in neurotransmission at the neuromuscular junction, and a reduction in muscle fibre size. These mechanisms align with the clinical presentation of hypotonia and muscle wasting observed in patients. MRI scans have revealed varying degrees of cerebral atrophy and demyelination, while muscle biopsies frequently show small fibres and selective atrophy of type II muscle fibres. EMG studies often detect abnormalities indicative of spinal motor neuron involvement [10].

In a recent review, Selamioglu et al. (2023) describes 10 patients with haemolytic anaemia diagnosed in newborns or the first months of age, preceding the neurologic symptoms [5]. It was also the case of our patient, as after an initial and isolated event of haemolytic anaemia at 2 months, she developed predominantly distal muscle weakness and hypotonia and progressive respiratory failure, with extensive control with NIV dependence. Selamioglu et al. reported a series of respiratory failure in 70% of patients older than this case. The neurologic compromise described is not homogeneous, with distal muscular weakness described in only two patients and progressive muscular dysfunction. The neuromuscular presentation of the patient described here is compatible with peripheral neuropathy, without severe axial weakness, and she is making neuromotor acquisitions. By nerve biopsy, Wilmshurst et al. (2004) demonstrated that peripheral neuropathy occurs in TPI deficiency [7]. Her previous neurodevelopmental status was normal and she seemed to have normal cognition.

Given that it was haemolytic anaemia with a negative DAT, a study of erythrocyte membrane and enzymatic disorders was conducted and subsequently excluded as part of the differential diagnosis. Haemoglobin levels remain stable, without blood transfusion, as described in a case reported by Sarper et al. [4].

An extensive work out on metabolic disorders was performed, looking for cobalamin and folate deficiencies and mitochondrial diseases due to the clinical presentation and the presence of megaloblastic anaemia, all of which were subsequently excluded. In this case, there was an increase in short-chain acylcarnitines, whereas the literature describes an isolated rise in C3 in patients with TPI deficiency. A possible explanation for this discrepancy could be secondary muscle injury (skeletal), which leads to increased free carnitine and, consequently, in short-chain acylcarnitine conjugates. The detection of elevated short-chain acylcarnitines, particularly C2, C3, and C5, in our patient warrants further consideration. While an isolated elevation in C3 has been previously reported in TPI deficiency, the additional elevations of C2 and C5 are not commonly described in the literature. These abnormalities may reflect secondary mitochondrial dysfunction or muscle catabolism, as the patient exhibited marked hypotonia and muscular atrophy. In this context, the acylcarnitine profile may reflect broader metabolic stress, possibly linked to defective energy metabolism in skeletal muscle. Although non-specific, these unusual findings reinforce the utility of acylcarnitine analysis in the diagnostic approach to neurometabolic conditions and may offer supportive biochemical evidence when evaluating suspected cases of glycolytic enzymopathies such as TPI deficiency. Future studies should investigate whether broader acylcarnitine disturbances may serve as early metabolic signatures in patients with TPI deficiency or related glycolytic defects.

A neuromuscular genetic panel was negative, and the association of haemolytic anaemia and neuromuscular symptoms elicited the diagnostic hypothesis of TPI deficiency, justifying the WES in a trio test. Two heterozygous missense variants, both classified as probably pathogenic in the *TPI1* gene, were identified, leading to the diagnosis of TPI deficiency. To our knowledge, this specific combination has not been previously described in the literature. Taken together, the identification of novel genetic findings alongside a broader acylcarnitine disturbance provides new insights into the pathophysiology of TPI deficiency and highlights the importance of integrating genetic and biochemical data when assessing suspected cases.

Most patients previously described do not survive beyond the age of six due to respiratory failure, the primary cause of death [8]. Nine months after diagnosis, our patient maintains significant respiratory dependence and requires feeding by nasogastric tube, although relevant motor progress. The mechanism of respiratory dependence in these patients is not discussed in the literature, although, at least in our patient, it is probably related to diaphragmatic paresis.

Presently, no specific treatment is available for TPI deficiency: enzyme substitution therapy, gene therapy and bone marrow transplantation are possible therapeutic strategies. Ationu et al. showed a significant increase in lymphocyte *TPI* activity and a reduced DHAP concentration following red-cell transfusion in an affected patient. This indicates that transferring functional enzymes to deficient cells and reversing metabolic blocks in glycolysis is feasible in vivo. Still, until now, no enzyme treatment has been produced [11].

In the context of personalised medicine, early genetic diagnosis not only confirms the aetiology but also provides prognostic information and allows for supportive strategies tailored to individual clinical features. The rarity of TPI deficiency presents significant challenges for both diagnosis and management. Moreover, uncertainty regarding the prognosis persists, as the progression of the disease can vary significantly between patients, making it difficult to predict clinical outcomes and long-term life expectancy. The limited understanding of the disease’s underlying mechanisms further hampers the development of effective therapies. As a severe paediatric condition without specific treatment, TPI deficiency requires primarily supportive care, such as feeding by nasogastric tube or gastrostomy and respiratory support. Further research into the disease’s pathogenesis is crucial to pave the way for more targeted treatments.

This case presents several novel aspects that contribute to the current understanding of TPI deficiency. Notably, the combination of elevated acylcarnitines C2, C3, and C5 is not previously reported in the literature as a consistent metabolic profile in TPI deficiency. This finding may represent a biochemical signature of mitochondrial dysfunction secondary to glycolytic blockade. Furthermore, the combination of two specific TPI1 variants observed in this patient has not been described in trans in prior case reports. These findings suggest a potentially distinct phenotype and support the expansion of the known biochemical and clinical spectrum of the disease.

## 4. Conclusions

This case highlights the complexities of diagnosing and managing TPI deficiency. It underscores the importance of multidisciplinary care in addressing the diverse challenges the disease poses and the need for ongoing research to improve outcomes for affected patients. Future studies should aim to establish genotype–phenotype correlations, identify early biomarkers of disease progression, and evaluate new therapeutic strategies—such as gene therapy or enzyme stabilisation—in pre-clinical models. Moreover, the growing accessibility to next-generation sequencing technologies may soon enable earlier diagnosis and better patient stratification, which will be essential for future clinical trials targeting ultra-rare metabolic diseases.

## Data Availability

The original contributions presented in this study are included in the article. Further inquiries can be directed to the corresponding author.

## Abbreviations

The following abbreviations are used in this manuscript:

ACMG	American College of Medical Genetics and Genomics
ATP	Adenosine Triphosphate
DAT	Direct Antiglobulin Test
DHAP	Dihydroxyacetone Phosphate
DTR	Deep Tendon Reflexes
EMG	Electromyography
G3P	Glyceraldehyde−3-Phosphate
MRI	Magnetic Resonance Imaging
NIV	Non-Invasive Ventilation
RSV	Respiratory Syncytial Virus
TPI	Triosephosphate Isomerase
TPI1	Triosephosphate Isomerase 1 gene
WES	Whole-Exome Sequencing
